# Adaptive myelination from fish to man

**DOI:** 10.1016/j.brainres.2015.10.026

**Published:** 2016-06-15

**Authors:** Marion Baraban, Sigrid Mensch, David A. Lyons

**Affiliations:** Centre for Neuroregeneration, University of Edinburgh, 49 Little France Crescent, Edinburgh EH16 4SB, UK

## Abstract

Myelinated axons with nodes of Ranvier are an evolutionary elaboration common to essentially all jawed vertebrates. Myelin made by Schwann cells in our peripheral nervous system and oligodendrocytes in our central nervous system has been long known to facilitate rapid energy efficient nerve impulse propagation. However, it is now also clear, particularly in the central nervous system, that myelin is not a simple static insulator but that it is dynamically regulated throughout development and life. New myelin sheaths can be made by newly differentiating oligodendrocytes, and mature myelin sheaths can be stimulated to grow again in the adult. Furthermore, numerous studies in models from fish to man indicate that neuronal activity can affect distinct stages of oligodendrocyte development and the process of myelination itself. This begs questions as to how these effects of activity are mediated at a cellular and molecular level and whether activity-driven adaptive myelination is a feature common to all myelinated axons, or indeed all oligodendrocytes, or is specific to cells or circuits with particular functions. Here we review the recent literature on this topic, elaborate on the key outstanding questions in the field, and look forward to future studies that incorporate investigations in systems from fish to man that will provide further insight into this fundamental aspect of nervous system plasticity.

*This article is part of a Special Issue entitled SI: Myelin Evolution*.

## Highlights

• CNS myelination by oligodendrocytes is a process that continues through adult life.
• Neuronal activity regulates many aspects of oligodendrocyte development.
• CNS myelination is adaptable and is also regulated by neuronal activity.
• Adaptive myelination can in turn affect nervous system function.

## 1 Introduction

The presence of myelin on axons greatly accelerates nerve impulse conduction (Rushton, 1951) and it is now clear that myelin plays additional important roles including the provision of metabolic support to axons that ensures their long-term health and survival (Fünfschilling et al., 2012; Lee et al., 2012b), as reviewed elsewhere in this issue. Over the past few years it has also become very clear that, in the central nervous system in particular, myelin is dynamically regulated throughout life. New myelin is generated in humans through adulthood and this is thought to be due to a combination of newly differentiating oligodendrocytes and remodeling of existing myelin sheaths (Yeung et al., 2014). Work in experimental models supports the premise that new myelin derives from both the generation of new myelinating oligodendrocytes through life (Hughes et al., 2013, Young et al., 2013) as well as the ability of mature myelin sheaths to have their growth reactivated in the adult (Snaidero et al., 2014). Furthermore, it has long been known that Central Nervous System (CNS) myelin can be replaced following damage, e.g. in conditions such as multiple sclerosis (MS), and that these myelin sheaths are made by newly differentiating oligodendrocytes (Zawadzka et al., 2010). However, it remains unclear what signals between axons and oligodendrocytes regulate the formation, remodeling or rejuvenation of myelin throughout life.

The concept that the actual activity of neurons and axons might itself regulate myelination has been in the literature for over fifty years (Gyllensten and Malmfors, 1963). Recently the study of the relationship between neuronal activity and the regulation of myelination has undergone something of a renaissance, due to the emergence of new technologies and models that allow ever more sophisticated means to manipulate nervous system function and assess consequent effects in real time and at high resolution. Beyond regulation of whether an axon is myelinated or not, the parameters of individual myelin sheaths also regulate conduction properties. For example increases to the length of individual myelin sheaths (also referred to as “internodes” in the literature, in reference to their location between consecutive nodes of Ranvier along axons) have been shown to cause an increase in conduction velocity, but only to a point, after which further increases do not cause further increase in speed (Huxley and Stampfli, 1949, Wu et al., 2012), and minor changes in the thickness of myelin relative to axon caliber (diameter) are known to elicit profound effects on conduction velocity (Smith and Koles, 1970). How other features of myelinated axons, e.g. node of Ranvier length (Moore et al., 1978), or longer unmyelinated gaps between consecutive myelin sheaths could be regulated to fine tune conduction remain untested experimentally. How neuronal activity precisely regulates any of the key features of myelinated axons is only now becoming clear. However, at the level of an entire system, whether the functional requirement is as “simple” as escaping a predator as fast as possible, or playing flight of the bumblebees on a piano at breakneck pace, it is entirely likely that activity-based regulation of myelinated axon structure is key to optimizing function. It is possible that activity regulates nervous system function by modulating the conduction properties of single neurons/ axons or indeed the coordinated function of neuronal ensembles (Pajevic et al., 2013, Seidl, 2014). However, numerous questions remain: Which aspects of axonal or oligodendrocyte development and myelination can be regulated by neuronal activity? Do all axons have the capacity to adaptively modulate their myelination status in response to neuronal activity? Do all oligodendrocytes have the capacity to adaptively regulate their myelinating behavior in response to changes in neuronal activity? Are there specific brain areas, or circuits that have a greater or lesser propensity for adaptive myelination? Does this ability change with age or in disease?

Interestingly, although new myelin can be made throughout life, how this is generated may differ between brain areas. While the frontal cortex of humans shows significant generation of new oligodendrocytes throughout life concomitant with increasing myelination, the corpus callosum in contrast shows little evidence of new oligodendrocyte generation, despite increasing its myelin content over time, suggesting remodeling/additional growth of existing sheaths (Yeung et al., 2014). It is technically possible, although less likely that the generation of new sheaths can be driven by existing oligodendrocytes (compare (Czopka et al., 2013; Makinodan et al., 2013; Watkins et al., 2008)). The myelination of axons of specific cortical neuronal subtypes can also be distinct, with some (but not all) axons displaying highly discontinuous myelination, whereby myelin sheaths are interspersed by large unmyelinated stretches, perhaps allowing for more function-driven adaptation of myelin sheath growth (Tomassy et al., 2014). These observations further suggest that specific brain areas, neuronal types, or circuits, may have more, or at least different, capacities for adaptive myelination than others. One intuitive prediction might be that those areas that are last myelinated in the animal, i.e. pre-frontal cortex, which are also involved in so-called “higher-order” functions may have the greatest capacity for adaptability. However, recent studies using the zebrafish as a model organism contradict the simplicity of this premise, whereby reticulospinal axons in the embryonic zebrafish spinal cord exhibit activity-regulated myelination (Hines et al., 2015; Mensch et al., 2015)). Reticulospinal neurons integrate sensory information to co-ordinate motor output, have the first myelinated axons in the zebrafish spinal cord (Almeida et al., 2011), and control the characteristic escape response (O׳Malley et al., 1996). That this “simple” system has the capacity for activity-driven adaptive myelination strongly suggests that the effect of neuronal activity on OL development and myelination may be more conserved across species, cells and circuits than intuitively assumed. Indeed, studies to date using zebrafish as a model demonstrate a high degree of molecular and cellular conservation with mammals with respect to myelination (Czopka and Lyons, 2011, Lyons and Talbot, 2014, Monk and Talbot, 2009). Therefore the premise that functional mechanisms should be concomitantly conserved is reasonable. Nonetheless, the degree of adaptability, and the likely diversity of neuronal and glial roles in adaptive myelination have barely been considered to date either within or between species, and thus warrant further consideration.

In this review we will focus on what recent studies in models from fish through man have taught us about the ability of myelinating glia to respond to neuronal activity, and how such adaptive myelination in turn affects nervous system function. We will focus on the CNS where most recent studies have been carried out and ask how neuronal activity can affect various stages of the oligodendrocyte lineage, from effects on early progenitors through the most mature myelinating cells. We will first summarize new insights into how neuronal activity regulates the early development of oligodendrocyte precursor cells (OPCs) (also called NG2 cells in the literature). We will then deal in detail with recent advances as to how activity affects the myelinating behavior of oligodendrocytes from a cellular and then molecular point of view. We will finally discuss how the environment regulates myelination and how, in turn, changes in myelination might regulate behavior. To date, different experimental models have provided insights into distinct aspects of activity-regulated adaptive myelination, due to complementary strengths and advantages, but we envision future research employing multiple evolutionarily diverse models to gain a better and more integrated understanding of this fundamental form of nervous system plasticity.

## 2 How does neuronal activity regulate oligodendrocyte development prior to myelination?

During embryonic development oligodendrocyte progenitor cells are specified in specific domains along the neuraxis, including the pMN domain in the ventral spinal cord, where they have been most extensively characterized. The morphogen-driven specification of neuroepithelial progenitor cells in the spinal cord is conserved across species and leads to the regulation of key transcription factors that specify oligodendrocyte fate. These have been reviewed extensively elsewhere and will not be dealt with in detail here (e.g. Mitew et al., 2013). Following delamination from the neuroepithelium, OPCs colonise the CNS by migration and proliferation and ultimately give rise to a stable homeostatically regulated population of cells that comprise 3-8% of all cells in adult the CNS (reviewed recently by (Dimou and Gallo, 2015)). In so doing, and depending on territory and indeed species, OPCs may increase in number over many orders of magnitude and migrate over extended territories. Seminal studies, over 20 years ago, first implicated neuronal activity as a regulator of the expansion of OPC number by regulating their proliferation, and more recent studies have started to elucidate the underlying mechanisms.

## 3 How does neuronal activity regulate oligodendrocyte precursor cell proliferation?

25 years ago several neurotransmitter receptors were found to be present and functional on glial cell progenitors in vitro (Barres et al., 1990), findings soon thereafter corroborated in oligodendrocytes analysed in brain slices of the mouse corpus callosum (Berger et al., 1992). Furthermore, neuronal activity was shown to profoundly regulate OPC number: when the functional activity of the developing optic nerve was blocked by axotomy or by treatment with the Na+ channel blocker tetrodotoxin (TTX) a striking decrease in OPC proliferation was observed (Barres and Raff, 1993). Until recently it was not clear whether this effect was likely to be mediated directly, indirectly, or even through neurotransmitters or alternative secreted factors.

**Fig. 1:**
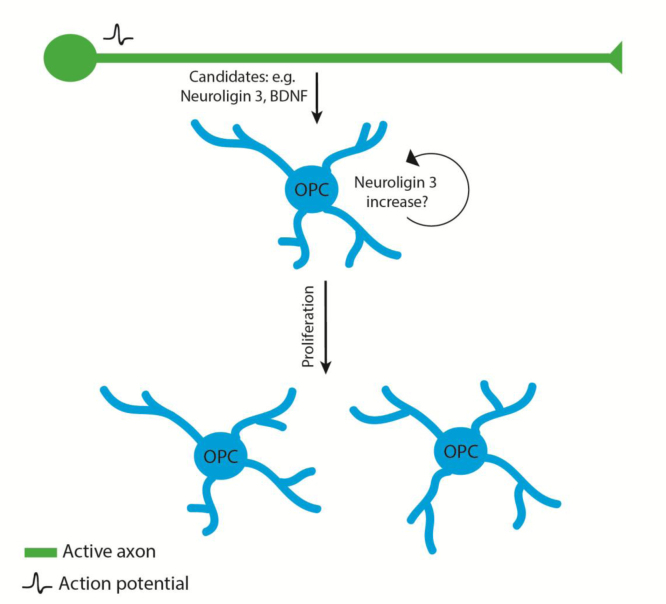
Model of how neuronal activity can lead to increased OPC proliferation. Activity mediated secretion of an unknown neuronal factor (possibly BDNF or Neuroligin 3 itself) leads to an increase in Neuroligin 3 expression in OPCs. This induces positive feed-forward signaling in OPCs, which in turn leads to increased proliferation.

Taking advantage of optogenetic technology (Deisseroth, 2011) to hyperactivate specific cortical neurons in living mice and analyse the effect on the oligodendrocyte lineage two related studies have begun to provide long-needed insight into the role of neuronal activity on OPC proliferation. Neurons in the premotor (M2) cortex of transgenic mice expressing the light-sensitive ion channel Channel Rhodopsin 2 were unilaterally stimulated at 20 Hz for 30 sec every 2 min for a 30 min period. This stimulation paradigm (and other variants) induced a characteristic unidirectional circular ambulation, and a striking increase in the proliferation of neuronal progenitor cells (NPC) and OPCs, confirming the previously implicated role of activity in inducing OPC proliferation (Gibson et al., 2014). Importantly, non-physiological activity, i.e. the induction of seizures, did not give rise to the proliferative response. In an extensive follow-up study using essentially the same optogenetic stimulation pattern on mice with engrafted cells derived from high-grade glioma (HGG), a similar effect was observed on proliferation (Venkatesh et al., 2015). Using biochemical approaches to identify the factor(s) mediating this effect of neuronal stimulation on glioma cell proliferation, Venkatesh et al concluded that the effect was mediated by a secreted protein between 10 and 100 kDa and mass spectrometry and functional investigations identified Neuroligin 3 as a major regulator (Venkatesh et al., 2015). Indeed activity-induced secretion of Neuroligin 3 (from an unknown source) induced further Neuroligin 3 expression in the glioma cells, further driving proliferation. At present it remains unclear precisely whether Neuroligin 3 is first secreted from neurons, axons, pre or post-synaptic compartments, or indirectly from other cell types including OPCs. This same study also made it clear that Neuroligin 3 is not the only activity regulated factor capable of stimulating glial cell proliferation. Indeed Venkatesh et al showed that although depletion of Neuroligin 3 from conditioned medium partially depleted the proliferative effect on HGG cells, this was more fully depleted by combined blockade together with a pharmacological inhibitor of TrkB, a receptor for the neurotrophin BDNF, which was also identified in the same study as a factor secreted following optogenetic stimulation (Venkatesh et al., 2015) (Fig. 1).

This study did not investigate the role of neurotransmitters in mediating the effect of activity on proliferation, and it is possible that neurotransmitter release initiates a cascade that activates OPCs to induce expression of Neuroligin 3, which kick-starts the feed-forward proliferation. Indeed, although Neuroligin 3 is highly expressed by neurons, it is actually most abundantly expressed by OPCs in the CNS (Zhang et al., 2014). The role of Neuroligin 3 in non-pathological oligodendrocyte development or myelination remains to be fully elucidated, but a recent study has indicated a role for this factor in oligodendrocyte differentiation (Proctor et al., 2015). However, early studies suggested that glutamate receptor activation in OPCs may actually negatively affect proliferation (Gallo et al., 1996, Yuan et al., 1998) and instead regulate the transition to differentiation, something further underscored by recent investigations of the role of activity and glutamatergic siganlling during remyelination (Gautier et al., 2015) (as described in the next section). Therefore, the precise role(s) of neurotransmission in OPC proliferation in vivo remains to be fully elucidated. It is also important to point out that OPC proliferation is regulated, likely in parallel, by other key mitogens such as platelet derived growth factor (van Heyningen et al., 2001), and by mutual interactions between oligodendrocytes themselves (Hughes et al., 2013, Huxley and Stampfli, 1949), the in depth discussion of which is beyond the scope of this review.

## 4 Neurotransmitter signaling and the axon-OPC synapse

Fifteen years ago identification of *bona fide* synapses between axons and OPCs was made– a landmark study in the field. In this work, stimulation of excitatory axons in the hippocampus was shown to depolarize associated OPCs, and this was revealed to be mediated by vesicular release of glutamate from axons signaling through calcium-permeable AMPA receptors on OPCs (Bergles et al., 2000). Furthermore this study demonstrated that sites of axon-OPC contact bore the ultrastructural hallmarks of traditional axon-dendritic synapses (Bergles et al., 2000). Two later parallel studies showed that such axon-OPC glutamatergic synaptic communication was not limited to the gray matter but also prominent in the white matter and could occur along the length of unmyelinated axons (Kukley et al., 2007, Ziskin et al., 2007). In addition to glutamatergic signaling, synapses between GABAergic axons and OPCs have also been documented and the axon-OPC synapse has been reviewed in and of itself extensively elsewhere (Almeida and Lyons, 2014, Bergles et al., 2010).

**Fig. 2:**
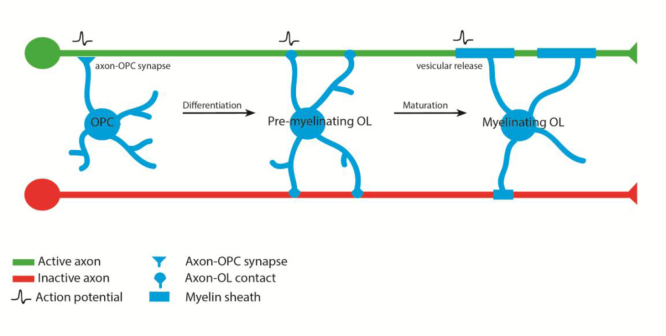
**:** Neuronal activity influences oligodendrocyte development during OPC differentiation and during myelination. Axon-OPC synapses are found between active axon and OPC processes and may regulate differentiation of OPCs to pre-myelinating/ immature oligodendrocytes. Pre-myelinating/ immature oligodendrocytes contact multiple axons prior to myelination. Activity-dependent vesicular release regulates the stability of nascent axon-OL contacts with specific targets and thus the likelihood of conversion of a contact into a mature sheath.

Recent studies have suggested that Glutamatergic (Mangin et al., 2012) and GABAergic (Zonouzi et al., 2015) synaptic inputs to OPCs can lead to changes in cell behavior. Mangin et al., found that stimulation of neurons in the ventrobasal nucleus directly induced synaptic currents in layer IV barrel cortex OPCs. Interestingly sensory deprivation of such thalamocortical input resulted in a change in OPC proliferation and distribution, although whether the phenotypic effects were directly or indirectly mediated by synaptic activation remains unclear (Mangin et al., 2012). More recently, a comprehensive study of the effects of hypoxia on OPC development indicated that the role of GABAergic input on to OPCs may be to regulate the transition from proliferation to differentiation, and that neurotransmitter based signaling positively drives differentiation and negatively regulates proliferation (Zonouzi et al., 2015). Indeed a recent study of the role of activity and glutamatergic signaling during remyelination has shown that disruption to AMPA receptor function impairs remyelinaiton, likely by regulating the transition of OPCs from proliferation to differentiation (Gautier et al., 2015) (Fig. 2), although the role for AMPA-R during developmental and non-pathological oligodendrocyte development and myelination remains to be determined. A very recent study has indicated additional levels of complexity with respect to axon-OPC synapse organization and dynamic function. Using paired recordings between fast-spiking interneurons (FSI) and non-fast spiking interneurons (NFSI) onto OPCs in the cortex, and analyses of the subcellular localization of GABA-A receptors with gamma 2 subunits in OPC processes, Orduz et al., showed that FSI form numerous synapses with OPCs in proximal parts of the cell, where as NFSI form many fewer synapses, lack the gamma2 subunit and target more distal OPC processes. Interestingly peak connectivity of GABAergic input coincides with the transition to differentiation (Orduz et al., 2015, Vélez-Fort et al., 2010), further implicating highly regulated synaptic signaling in this key developmental transition between OPC and oligodendrocyte. There is also accumulating evidence that adult OPCs may differ, at least somewhat, in their functions compared to OPCs in developing animals (Maldonado et al., 2013, Sakry et al., 2014), and so the effects of neuronal activity on adult OPCs may be distinct from those on cells fated for differentiation and myelination during development.

Studying the transition from OPC proliferation to differentiation in vivo is problematic, because we know very little about the dynamic cell behavior of OPCs and oligodendrocytes in the living animal. A recent study of early postnatal myelination in rodents has suggested the existence of a critical period of a few days for differentiation following OPC division (Hill et al., 2014), although the timing of oligodendrocyte differentiation may be different in adults (Hughes et al., 2013). Indeed there is also evidence for a later, and even shorter, critical period (on the order of hours) for myelin sheath initiation by individual oligodendrocytes in both zebrafish and rodents (Czopka et al., 2013, Watkins et al., 2008), but how these two potentially distinct critical periods for the transition to differentiation and subsequent myelin sheath generation relate to one another is not clear. Previous studies in rodents have suggested that oligodendrocyte differentiation and myelination can be uncoupled (Rosenberg et al., 2007), and it is likely that neuronal activity affects both of these transitions independently (Fig. 2), the first from OPC to differentiated OL potentially through a synaptic mechanism, as discussed above, and the second to myelination, perhaps through a non-synaptic mechanism, as discussed below.

Sophisticated live imaging of the oligodendrocyte lineage is now possible in superficial layers of the adult rodent cortex using two-photon imaging (Hughes et al., 2013), which complements the natural imaging capacities of developing zebrafish, in which oligodendrocyte development and myelination can be monitored in the living embryo in real time using fluorescent reporters (Czopka et al., 2013, Kirby et al., 2006, Snaidero et al., 2014). Such imaging approaches in distinct species and at different stages of life will help bridge our understanding of the transition of cells of the oligodendrocyte lineage from OPC right through myelination in vivo.

## 5 How does neuronal activity regulate myelination?

Despite the highly regulated timing of myelination in vivo with respect to distinct areas of the CNS, or even distinct axons or tracts in the same area, there is an extensive body of evidence to suggest that myelination by oligodendrocytes may *require* very little in the way of instructive cues. For example, oligodendrocytes can myelinate paraformaldehyde fixed axons (Rosenberg et al., 2008) and even inert plastic fibers in vitro (Lee et al., 2012a). In fact a recent study has shown that oligodendrocytes derived from different areas of the CNS can generate myelin sheaths on inert plastic fibers of the same length as observed within the corresponding areas in vivo (Bechler et al., 2015), suggesting that even properties of individual myelin sheath growth may in principal be regulated independently of activity. Furthermore, numerous studies have characterized the electrophysiology of cells of the oligodendrocyte lineage and the emerging consensus is that synaptic currents diminish as cells differentiate and particularly as oligodendrocytes undergo myelination (Clarke et al., 2012, De Biase et al., 2010).

Despite the implications from such studies that myelination may receive little axonal regulation, previous studies have indicated that axons profoundly regulate myelinating behavior in vivo (Almeida et al., 2011), that expression of neurotransmitter receptors are expressed in myelinating cells (Butt et al., 2014, Karadottir et al., 2005, Micu et al., 2005, Salter and Fern, 2005), and that calcium activity can be detected in mature myelin (Micu et al., 2007), suggesting that oligodendrocytes remain responsive to neuronal activity at all stages. Numerous studies over the past fifty years have asked the question if and how neuronal activity might regulate myelination, which has been summarized in numerous reviews (Fields, 2014, Fields, 2010, Fields et al., 2014, Zalc and Fields, 2000). The consensus is that activity plays a key role in regulating myelination and that activity-regulated myelination may represent an important form of nervous system plasticity (Fields et al., 2014, Pajevic et al., 2013). This begs the question as to how one can reconcile the apparently inherent ability of oligodendrocytes to generate myelin (with parameters mirroring those in vivo) in the absence of active axons with the proposed role for neuronal activity in regulating myelination. New studies have helped unravel this paradox.

## 6 Activity-driven regulation of cellular mechanisms of myelination

Recently, two studies have investigated how neuronal activity and synaptic vesicle release affect CNS myelination in vivo using zebrafish as a model. Because of the limited expansion of the OPC pool and oligodendrocyte number prior to the first wave of myelination in the zebrafish spinal cord (Mensch et al., 2015), investigation of the role of activity on myelination can be carried out without the confounds of a significant effect on OPC proliferation. Furthermore, the exquisite properties of zebrafish for live imaging allow analyses of cell morphology and dynamics in ways that have been difficult to date in other systems. In the two recent studies of activity-regulated myelination in zebrafish, tetanus toxin, which cleaves the v-SNARE VAMP2/ synaptobrevin, was used to prevent synaptic vesicle release. In one study Mensch et al., globally expressed tetanus toxin and found about 40% fewer myelinated axons in the spinal cord at early larval stages and showed that this was specific to myelination, because the number and caliber of relevant axons was completely normal (Mensch et al., 2015). They showed that this reduction in the myelination of axons was primarily due to a reduction in the myelin sheath number of individual oligodendrocytes, a result mirrored in the second study by Hines et al., who expressed tetanus toxin in all neurons using a pan-neuronal driver (Hines et al., 2015). These studies indicate that vesicular release regulates the myelinating capacity of individual oligodendrocytes in vivo. It is noteworthy that in both of these studies, individual oligodendrocytes retained about 60% of the normal number of myelin sheaths, perhaps representing the previously noted basal/intrinsic myelinating capacity of oligodendrocytes that can occur independently of axonal signals. Importantly, Mensch et al., showed that increasing activity lead to individual oligodendrocyte generating about 40% more sheaths per cell than normal, in a vesicle release dependent manner, a result that further highlights the plasticity of the myelinating behavior of oligodendrocytes and their regulation by activity. Therefore we propose that it is more accurate to consider neuronal activity as a modulator or regulator of myelination, rather than as being *required* for myelination or speaking about activity-dependent myelination.

Time-lapse analyses of oligodendrocyte behavior in the presence and absence of synaptic vesicle release provided further insight into underlying mechanisms of how vesicular release regulated the myelinating behavior of oligodendrocytes. Mensch et al showed that the reduction in myelin sheath number per cell was already evident by the end of the characteristic short period of 4–6 h of myelin sheath initiation by individual oligodendrocytes. Hines et al. further refined these observations, and showed that oligodendrocyte processes initially formed the same number of contacts with axons irrespective of vesicular release, suggesting that activity independent mechanisms regulate initial axon-oligodendrocyte targeting prior to myelination. What Hines et al., observed, however, was that the stability and initial growth of such axon-OL contacts/ nascent myelin sheaths was greatly reduced within the first 90 min in the absence of vesicle release. These data indicate that vesicular release stabilizes such initial axon-OL contacts prior to their elaboration as mature myelin sheaths. Indeed, once nascent sheaths grew >10 µm in length their stability was the same in the presence or absence of vesicular release. In order to test whether the effect of activity on myelin sheath formation was likely due to vesicular release at sites of local axon-OL contact, both Mensch et al., and Hines et al., expressed tetanus toxin in individual neurons, and assessed myelination. In support of local axon to OL vesicular release the number of myelin sheaths along the length of single axons expressing tetanus toxin was reduced (Hines et al., 2015, Mensch et al., 2015). These data were recapitulated in a recent elegant in vitro study of mammalian myelination in which dorsal root ganglion and oligodendrocytes were co-cultured and it was found that axons with impaired vesicular release (due to pretreatment of neurons with Botulinum toxin) were myelinated much less frequently than active axons in the immediate proximity (Wake et al., 2015). Although this vesicular release-regulated myelination was shown to be mediated by glutamate release onto oligodendrocytes, no evidence of actual synaptic currents in oligodendrocytes was found (Wake et al., 2015). The authors did find evidence for calcium activity in oligodendrocytes, and ultrastructural evidence of axon-OL contacts without the hallmarks of mature synapses, implicating a non-synaptic mode of neurotransmitter release driving myelin sheath formation (Wake et al., 2015). Interestingly, in both the zebrafish in vivo and mammalian in vitro studies, a reduction in myelin sheath length was also observed along individual axons with impaired vesicular release, suggesting the possibility of independent roles for vesicular release in regulating sheath formation by stabilizing initial axon-OL contacts, and subsequent growth. Indeed, following their optogenetic stimulation of the motor cortex Gibson et al., also noted an increase in myelin thickness in axons of the corpus callosum, further indicating a potential later role for activity in regulating myelin sheath growth (Gibson et al., 2014), although whether this effect is directly regulated by individual axons remains unclear, as is the case for the intriguing reduction in myelin sheath thickness observed following disruption to activity during remyelination (Gautier et al., 2015) Future studies that manipulate neuronal activity at distinct stages in vivo and directly observe effects on cell behavior will be required to test how interactions between individual axons and associated oligodendrocyte processes regulate distinct aspects of myelination and remyelination in vivo.

## 7 How can myelination be adapted along single axons?

**Fig. 3:**
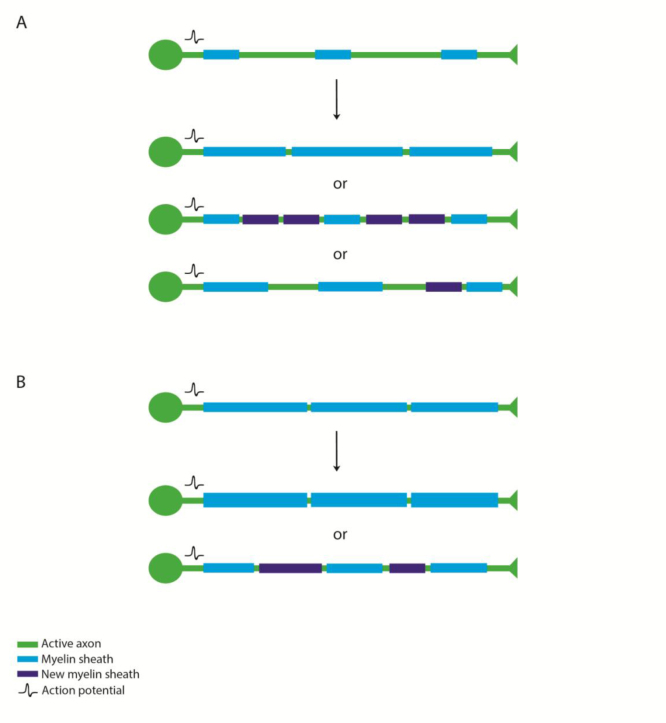
: Models of adaptive myelination in partially myelinated axons and fully myelinated axons. (A) Changes in neuronal activity in a partially myelinated axon could lead to the full myelination along the length of a partially myelinated axon by increasing myelin sheath length, by the addition of new myelin sheaths, or a combination of both. (B) Changes in neuronal activity along a fully myelinated axon could lead to increased myelin thickness or the intercalation of newly formed myelin sheaths.One assumes that if truly plastic, such patterns of myelination are reversible.

**Fig. 4:**
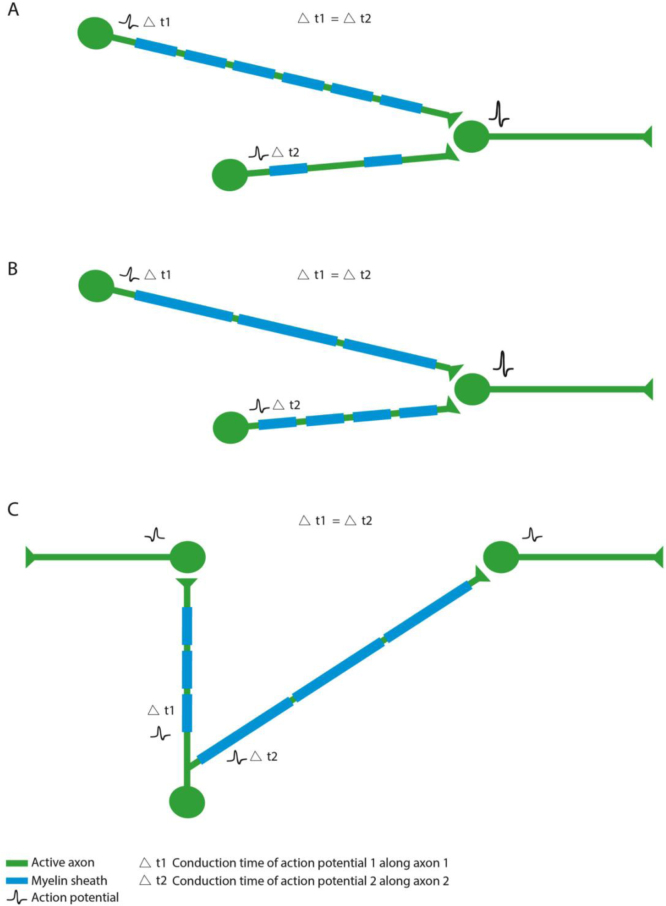
: Model of adaptive myelination in a neuronal circuit. In order to facilitate the synchronized arrival of two action potentials from two neurons with axons of different axon length, the neuron that is located closer to the postsynaptic site might have a partially myelinated (A) (or indeed unmyelinated axon), or have shorter or thinner myelin sheaths (B). Example of a single neuron that coordinate impulse arrival at targets at distinct distances apart (C).

Given the evidence that activity can regulate myelin sheath formation and growth, how might such myelin adaptation affect circuit function? Two recent reviews have articulated on how regulation of myelination affects the conduction properties of individual axons (Seidl, 2014) and might even regulate the emergent properties of neuronal ensembles (Pajevic et al., 2013). Therefore, we will focus instead on how from a cell and developmental point of view myelin along the length of axons might be regulated by activity/ vesicular release. During de novo myelination (by newly differentiating oligodendrocytes), new myelin sheaths can in principal be formed either around axons that were previously completely unmyelinated or axons that were previously, but incompletely, myelinated, or indeed to change the distribution of myelin sheaths along fully myelinated axons (Fig. 3). A recent study investigated diversity in myelination along individual axons by interrogating available 3D electron microscopy datasets of the rodent neocortex (Tomassy et al., 2014). In their analyses, Tomassy et al., showed that certain neurons (layer II–III cortical neurons) have axons that have variable degrees of myelination along their length, i.e. areas of myelination interspersed with very long areas without myelin (Tomassy et al., 2014). Although it is not clear how common this variable myelination is along different neuronal cell types or whether this remains true in adults, the principle that such axons may have more room for adaptive myelination stands. A previous analyses of the optic nerve indicated that conduction velocity may change along the length of the nerve (Baker and Stryker, 1990), and that this is most likely due to changing diameter of the myelinated axon along its length, another important parameter that may in principal be under regulation by neuronal activity. In addition to the de novo myelination of unmyelinated axons or unmyelinated areas of myelinated axons, new myelin sheaths might also replace old myelin sheaths or might be intercalated between existing myelin sheaths along an already fully myelinated axon, as has recently been proposed (Young et al., 2013) (Fig. 3). A fundamental feature of plasticity is that change is reversible: therefore it is important that we consider adaptations such as myelin sheath retractions and shrinking with respect to activity-driven regulation of myelination too. We have observed retraction of relatively mature myelin sheaths during normal nervous system development (Czopka et al., 2013), although it remains unclear if this is an activity regulated refinement along myelinated axons. There are numerous examples in the literature where fewer and shorter myelin sheaths are key to regulating nervous system function (Seidl, 2014), e.g. in coordinating the synchronous arrival of impulses at a target in the chick auditory brainstem, where impulses are initiated in neurons of variable distances from the target but arrive synchronously at their target (Seidl, 2014). Previous studies had suggested that the introduction of a delay line (i.e. simple lengthening of the axon of the neuron closer to the target by sending it on a circuitous journey) could allow for the synchronous arrival of impulses from neurons of different distances apart. However, careful anatomical reconstructions invalidated this hypothesis, at least for the chick acoustic system and instead showed that changes to the parameters of myelinated axons (axonal caliber and myelin sheath number length and thickness) could account for specific conduction times that would underpin synchronous arrival at coincident detector neurons (Seidl et al., 2010). Whether these interesting anatomical functional adaptations are activity driven remains to be determined. It is interesting to note that distinct myelination parameters thought to coordinate synchronous arrival from either hemisphere are actually made on different branches of single individual neurons (Fig. 4). It is hard to imagine how the firing pattern of a single neuron could adaptively and distinctly modulate the myelination of its different axonal branches, so one possibility is that feedback from target neurons can regulate axonal and myelin parameters, as has been shown in the peripheral nervous system (Voyvodic, 1989). Alternatively, it could be that the distinct axonal branches were formed and myelinated at distinct developmental periods and by oligodendrocytes of differing myelinating potential. Addressing these open issues will require taking an approach that integrates analysis of neuronal, axonal and myelinating glial cell development, as well as modulation.

## 8 Activity-driven regulation of molecular mechanisms of myelination

Despite the emerging consensus that neuronal activity can regulate myelin sheath formation, the underlying molecular mechanisms remain unclear. Vesicular release, most likely of of neurotransmitter, remains the primary candidate with respect to the axonal signal and two related in vitro studies have suggested that vesicular glutamate release from axons can stimulate the local translation of myelin basic protein (mbp) mRNA into protein in associated oligodendrocyte processes. Further pharmacological manipulations in the same DRG-oligodendrocyte co-culture system, implicated both NMDA and metabotropic glutamate receptors in oligodendrocytes in mediating this effect, and the Src family non-receptor kinase Fyn as the intermediary regulator of local *mbp* translation (Wake et al., 2011). Although subsequent in vivo analyses failed to show a significant effect of deletion of the obligate NMDAR subunit NR1 from the oligodendrocyte lineage on myelination (De Biase et al., 2011, Guo et al., 2012), the possibility of glutamate receptor redundancy cannot be excluded. Indeed, disruption of Fyn in vivo, in either zebrafish (Czopka et al., 2013) or mice (Umemori et al., 1994) does reduce CNS myelination, and its activation in myelinating oligodendrocytes in zebrafish increases the number of myelin sheaths produced by individual oligodendrocytes (Czopka et al., 2013), in a similar manner to increasing neuronal activity (Mensch et al., 2015). However, formal demonstration that these observations are mechanistically linked awaits testing.

Alternative/parallel models of how activity might regulate myelination have also been proposed. A recent model proposes that neuronal activity regulates Neuregulin 1 and/or BDNF release from axons to regulate NMDAR clustering in associated oligodendrocyte processes and thereby switch cells from an activity independent mode of myelination to an activity (NMDAR) dependent, or perhaps more specifically “regulated,” mode of myelination (Lundgaard et al., 2013). Although this hypothesis also awaits formal testing in vivo, it is of note that tetanus toxin sensitive VAMP2 vesicles can also release BDNF in an activity dependent manner (Shimojo et al., 2015), suggesting at least the formal possibility that BDNF may also be the operant factor mediating the tetanus toxin mediated phenotypes observed in zebrafish. Furthermore, specific firing rates can regulate Neuregulin expression on neurons, but how this relates to processing and secretion of the form that has been shown to regulate myelination in vitro remains to be determined. However, it is intriguing that Neuregulin-erbb signaling has been implicated in behaviorally relevant cortical myelination as discussed in a following section, implicating an activity-related role of this pathway in myelination.

Careful cell-type specific genetic manipulation, potentially of multiple neurotransmitter (and other) receptor subtypes in oligodendrocytes in parallel, and epistasis analyses of proposed downstream signaling pathways will be required to elucidate the molecular basis of activity-based regulation of the different stages of myelination.

## 9 How does activity-driven adaptive myelination regulate nervous system function?

Social isolation in humans, as well as rodent models, leads to severe impairments in learning and memory capacities, and recent evidence implicates disruption to CNS myelination as being a contributor to these phenotypes. In order to test how social isolation affects myelination, Makinodan et al., socially isolated cohorts of juvenile mice at specific periods. They found hypomyelination in the prefrontal cortex (PFC) of mice reared in isolation, whereby oligodendrocyte had a simpler morphology with reduced branch and myelin sheath number per oligodendrocyte and also reduced myelin thickness (Makinodan et al., 2012). They also observed concomitant deficiencies in a range of behavioral assays. Intriguingly, these myelin and behavioral effects were observed when social isolation took place during early development (4th and 5th week postnatal), but not when animals were isolated after the 6th week of life, suggesting a critical period during development when myelination of the PFC is plastic. Makinodan et al., further found that social isolation reduces the expression of neuregulin type III, and intriguingly that an oligodendrocyte-specific knockout of the Neuregulin receptor ErbB3 phenocopied both the behavioral deficits and hypomyelination induced by social isolation (Makinodan et al., 2012). A second independent study showed that prolonged social isolation for 8 weeks during adulthood could also lead to hypomyelination in the PFC of mice and that this was reversible, unlike the developmental effects of social isolation (Liu et al., 2012). Liu et al. further found a reduction in nuclear chromatin compaction and an increase in histone acetylation in the PFC in juvenile and adult mice, parameters associated with immature oligodendrocytes and impaired myelination (Liu et al., 2012), indicating that social experience can induce epigenetic changes in oligodendrocytes.

A separate study of adult rats indicated that animals housed in an enriched environment for a 4 month period showed increased myelination in the corpus callosum as well as improved spatial learning (Zhao et al., 2012). Although an effect of environmental enrichment was not observed on developmental myelination in the PFC (Makinodan et al., 2012), the duration of enrichment and distinct species mean that these are very difficult situations to compare. Nonetheless, together these studies indicate that the sensory environment plays important roles in myelination and cognition, and one assumes this is driven by regulation of neuronal activity. Whether only specific forms of experience can modulate myelin and whether this is a specific feature of distinct brain areas or at distinct times remains to be determined.

Although these studies correlate changes in environmental input with myelination and changes in myelination with behavior, they do not directly test the role for myelination in regulating behavior. However, a landmark study in the field recently addressed this experimental gap. In a motor learning model, in which mice run on a complex running wheel with irregularly spaced rungs and therefore have to learn a new mode of running, McKenzie et al. observed a transient increase in OPC proliferation and oligodendrocyte differentiation in the corpus callosum after a brief learning period (McKenzie et al., 2014). In order to begin to analyze the contribution that oligodendrocytes and myelin have on cognitive functions, such as learning, McKenzie et al. (2014) inhibited the formation of new myelinating oligodendrocytes in the adult mouse brain. Through tamoxifen induced Cre-mediated homologous recombination, McKenzie et al. were able to conditionally knock-out the myelin regulatory factor (Myrf), which is required for the differentiation of mature myelinating oligodendrocytes (Emery et al., 2009), specifically in OPCs. Crucially this manipulation allowed the authors to disrupt the generation of new adult-born oligodendrocytes without affecting preexisting oligodendrocytes or preexisting myelin. Through this strategy, they were able to show that mice without the ability to generate newly differentiated oligodendrocytes had marked defects in their ability to learn to adapt to the complex running wheel task (McKenzie et al., 2014). Importantly, when mice learned to run on the complex running wheel before Myrf knock-out, they performed similarly to control mice. This indicates firstly, that the Myrf knock-out did not impair the overall motor function of these mice and secondly, that newly formed oligodendrocytes and myelin are not required for the retrieval of a pre-learnt motor skill but regulate learning the new motor task. This study therefore elegantly demonstrates that the differentiation of oligodendrocytes, and *presumably* the generation of new myelin (this was not yet formally shown), is essential for learning complex new motor skills. Precisely which neuronal circuits acquire new myelin, or how myelin might be remodeled during such learning remains unknown. Examining such adaptive myelination from the perspective of circuits will be important in future studies.

## 10 “White matter” changes during learning

Approximately 50% of the human brain by volume is composed of “white matter.” White matter is largely composed of myelinated axons, but it is important to note that there is significant myelination throughout the grey matter, and that white matter also contains an abundance of astrocytes and vasculature (Zatorre et al., 2012). Brain imaging studies based on Magnetic Resonance Imaging (MRI) often assess changes in the context of grey and white matter, and alterations to grey matter have been well correlated with learning (Zatorre et al., 2012). Increasingly, however, it is now clear that white matter changes are apparent in the context of learning and attempts have been made to correlate changes in “white matter” with cellular underpinnings. Through assessment of fractional anisotropy (FA) values following MRI, one can define a proxy of white matter structure and organization in the CNS. White matter changes observed in the corpus callosum following learning of a spatial navigation task were correlated with increases in Myelin Basic Protein (MBP) expression in the corpus callosum, indicating that increased myelination could, at least in part, account for the white matter changes (Blumenfeld-Katzir et al., 2011). However, adult neurogenesis has also been observed during hippocampal dependent acquisition of new motor skills (Gould, 1999) and the increase in MBP expression in the corpus callosum might be caused by the myelination of newly formed axons. In order test how myelin changed in response to learning a new motor skill, Sampiao-Baptista et al., investigated white matter plasticity in the cortex, where neurogenesis in the adult is largely absent. In a skilled reaching assay, rats learned a novel motor skill in which they were trained to reach and acquire a sugar pellet with one of their paws. After successful acquisition of that skill, the white matter underlying the forelimb motor cortex was compared to control groups, which had not learned the new motor skill. Sampiao-Baptista et al. found a significant increase in FA in the animals that had learned the skilled reaching technique in the contralateral hemisphere to the trained paw, but not in the ipsilateral hemisphere, demonstrating white matter plasticity can occur (likely independently of neurogenesis) and that it is specific to the cortical areas to which the trained paw projected (Sampaio-Baptista et al., 2013). Furthermore, a positive correlation between FA and the learning rate of the individual rats in acquiring the novel motor task was found, indicating that the rate with which a novel task is acquired is associated with the underlying white matter changes. By combining MRI imaging with immunohistochemistry, they found that MBP stain intensity was also significantly increased in the cortical areas contralateral, but not ipsilateral, to the trained paw of animals that was used during learning of the new motor task. Interestingly, however, a statistically significant correlation between MBP and FA was not found. This could be due to a small sample size, but might also indicate that an increase in myelination alone is not the only factor accounting for white matter changes. This apparent disconnect highlights a gap in our understanding of white matter changes seen by MRI and the underlying cellular and sub-cellular correlates. It is important to note that changes in myelination are likely to represent only a component of white matter adaption. Other prospective adaptive changes to white matter include modulation of axonal caliber, axonal organization, and even dynamic regulation of other cell types including astrocytes or cells of the nervous system vasculature (Zatorre et al., 2012). To what degree myelin adaption plays a primary role in white matter plasticity remains to be determined. Future studies that directly compare white matter signatures with high-resolution brain area and circuit reconstruction such as those that can now be provided by 3D reconstruction by light sheet and electron microscopy will be required to bridge this gap.

## 11 White matter changes in humans during learning

White matter alterations after acquisition of a novel motor learning task has also been shown in humans. Learning a novel visuo-motor task such as juggling for only a 6 week period (Scholz et al., 2009) has been shown to change the structural integrity of white matter in the intraparietal sulcus, a brain area associated with visuo-motor skills. Furthermore, increased white matter in brain regions associated with sensory-motor processing has been shown in humans after performing specific tasks such as piano practice (Bengtsson et al., 2005), and Baduk playing (Lee et al., 2010). Interestingly, the amount of white matter positively correlated with the practicing time (albeit self-reported for pianists) and occurred in different brain regions, depending on the age during which the practice took place (Bengtsson et al., 2005). While childhood practicing resulted in increased white matter in the bilateral posterior limbs of the internal capsule as well as in the corpus callosum, piano practice during adolescence correlated with a white matter increase in the corpus callosum and the splenium, extending into the white matter of the occipital lobe. Adult practicing, in contrast correlated with increase white matter in the left anterior limb of the internal capsule and the right temporoparietal junction (Bengtsson et al., 2005). These differences in neuronal activity-driven white matter alterations again suggest that neuronal activity may regulate white matter structure and function in region and stage specific manners, posing the question as to whether adaptive myelination is favored or even restricted to periods when specific brain areas are still undergoing “developmental” myelination. Considering that humans myelinate new brain areas well in to adult life (Wang and Young, 2014), the concept of “developmental” myelination has a rather broad meaning. Furthermore, it will be important in the future, to consider whether these alterations in white matter are reversible, and if so, how this relates to function. Future studies comparing adaptability throughout life and into old age, will continue to elucidate the dynamics of adaptive myelination over time.

## 12 Conclusion

Following fifty years of investigation, it is now abundantly clear that neuronal activity plays a key role in the regulation of CNS myelination by oligodendrocytes. It is also becoming clear that such activity-driven myelin regulation is important in learning and memory. What remains to be elucidated are the underlying molecular mechanisms, which the research community is well poised to do, and also the circuit specific regulation of myelination, particularly over time. It is entirely possible that myelination may be the one of most adaptable parameters in the mature CNS, particularly given the preponderance of OPCs in the adult brain and the ability of the brain to repair damaged myelin. It may even be conceivable of a future in which this adaptability can be co-opted to enhance normal nervous system function, in addition to regulating endogenous repair, as is envisioned for the treatment of diseases of myelin. Before that time comes, we look forward to studies using evolutionarily diverse models to tease apart the mechanisms underlying this important form of nervous system plasticity.
